# ROS-activated CXCR2^+^ neutrophils recruited by CXCL1 delay denervated skeletal muscle atrophy and undergo P53-mediated apoptosis

**DOI:** 10.1038/s12276-022-00805-0

**Published:** 2022-07-21

**Authors:** Yaoxian Xiang, Junxi Dai, Yao Li, Zongqi You, Junpeng Zhang, Xinying Huang, Shuqi Nie, Yujie Chen, Lei Xu, Fengming Liu, Junjian Jiang, Jianguang Xu

**Affiliations:** 1 Department of Hand Surgery, Huashan Hospital, Fudan University, Shanghai, People’s Republic of China; 2 NHC Key Laboratory of Hand Reconstruction (Fudan University), Shanghai, People’s Republic of China; 3 Shanghai Key Laboratory of Peripheral Nerve and Microsurgery, Shanghai, People’s Republic of China; 4 National Facility for Protein Science in Shanghai, Zhangjiang Lab, Shanghai Advanced Research Institute, Chinese Academy of Sciences, Shanghai, 201210 People’s Republic of China; 5 School of Rehabilitation Science, Shanghai University of Traditional Chinese Medicine, Shanghai, People’s Republic of China; 6 Shanghai Medical College of Fudan University, Shanghai, People’s Republic of China

**Keywords:** Neutrophils, Chemokines

## Abstract

Neutrophils are the earliest master inflammatory regulator cells recruited to target tissues after direct infection or injury. Although inflammatory factors are present in muscle that has been indirectly disturbed by peripheral nerve injury, whether neutrophils are present and play a role in the associated inflammatory process remains unclear. Here, intravital imaging analysis using spinning-disk confocal intravital microscopy was employed to dynamically identify neutrophils in denervated muscle. Slice digital scanning and 3D-view reconstruction analyses demonstrated that neutrophils escape from vessels and migrate into denervated muscle tissue. Analyses using reactive oxygen species (ROS) inhibitors and flow cytometry demonstrated that enhanced ROS activate neutrophils after denervation. Transcriptome analysis revealed that the vast majority of neutrophils in denervated muscle were of the CXCR2 subtype and were recruited by CXCL1. Most of these cells gradually disappeared within 1 week via P53-mediated apoptosis. Experiments using specific blockers confirmed that neutrophils slow the process of denervated muscle atrophy. Collectively, these results indicate that activated neutrophils are recruited via chemotaxis to muscle tissue that has been indirectly damaged by denervation, where they function in delaying atrophy.

## Nerve injury: Immune cells defend against muscle wasting

Live animal imaging experiments reveal how rapid recruitment of a subset of immune cells helps prevent muscle wasting after peripheral nerve injury. Such injuries take considerable time to heal, and there are no therapies that reliably prevent wasting of muscle lacking nervous innervation. Researchers led by JunJian Jiang and Jianguang Xu at Fudan University, Shanghai, China, have used intravital microscopy to record the cellular and molecular events that follow nerve damage in live mice. They observed heightened production of chemicals that summon immune cells known as neutrophils to the site of the injury. Even though the surrounding muscle cells were initially undamaged in this animal model, the recruited neutrophils delayed subsequent muscle wasting. This neutrophil recruitment was transient, but therapies that elicit a more sustained response could provide durable protection against muscle wasting.

## Introduction

Regenerated nerve axons grow slowly after peripheral nerve injury, and the slow recovery of denervated muscle function is therefore of clinical concern^1–3^. As such, increasing effort has recently been focused on the development of effective treatments for nerve injury, with some success reported^4,5^. However, existing treatments for directly delaying or reversing muscle atrophy are inadequate. It is therefore critical to enhance the understanding of the pathological mechanism and microenvironment of denervated muscle tissue to develop more effective treatments.

Previous studies have found that macrophages, as skeletal muscle–resident innate immune cells, function to maintain tissue homeostasis and promote muscle growth and regeneration^6,7^. During inflammation, macrophages release a large number of proinflammatory factors^8,9^. In addition, neutrophils, which are not present in normal muscle tissue, are directly recruited to and localize in sites of tissue injury (such as infection-associated inflammation or sterile injury), where they also play a role in mediating acute inflammation and recovery processes^10–13^. This neutrophil infiltration process likely limits the innate immune response to areas of primary damage to protect adjacent healthy tissue^12,14^. However, in cases of peripheral nerve injury, the associated denervated muscle is not directly damaged. To our knowledge, as no studies demonstrating the presence of neutrophils in denervated muscle have been published to date, whether these cells participate in this type of aseptic inflammation after indirect damage is unclear.

Previous studies have found that the generation of reactive oxygen species (ROS) is an important step in neutrophil activation and migration across blood vessels in infectious inflammation^15,16^. Other studies found that excessive ROS generation is involved in the induction of muscle atrophy and weakness^17–20^. Denervation causes target muscle tissue to enter a state of relative ischemia and hypoxia, which in turn induces excessive ROS production and systemic stress injury^21–23^. These processes lead to the production of a large number of inflammatory factors and the expression of downstream atrophy-related genes^24,25^. Therefore, excessive ROS production creates the basic environmental conditions in denervated skeletal muscle tissue that are necessary for inflammation. However, whether neutrophils play a role in ROS-induced inflammation under these conditions remains unknown.

Several studies have suggested that neutrophil function is related to the intrinsic properties of neutrophil subpopulations^26,27^. Treatments that directly reduce or increase the number of neutrophils present, regardless of phenotype, may therefore inadvertently cause tissue harm^28,29^. Furthermore, the dominant chemokines of distinct functional neutrophil subtypes differ among disease models^30^. Thus, defining the neutrophil subtypes and identifying the particular chemokines are critical.

Tissue repair depends upon not only the early infiltration of neutrophils but also their timely resolution. Apoptotic death of tissue-infiltrating neutrophils is required for the rapid resolution of acute inflammation to restore tissue structure and function^31,32^. Inflammatory cytokines such as granulocyte-macrophage colony-stimulating factor and microbial constituents such as bacterial DNA (CpG DNA) extend neutrophil longevity in tissues by delaying apoptosis^33,34^. In contrast, other mediators, such as 15-epi-LXA4 and resolvin E1, ultimately stimulate neutrophil apoptosis, thus limiting the number of neutrophils at sites of inflammation and directing ongoing inflammation toward resolution^35,36^. Identifying apoptosis-related mediators could therefore be productive for facilitating the development of treatments for excessive inflammation.

In the present study, we used intravital imaging with spinning-disk confocal intravital microscopy (SD-IVM) to clearly demonstrate the dynamic behavior of neutrophils inside the vessels of denervated muscle. The spatial distribution of neutrophils and blood vessels in denervated muscle was determined using 3D-view reconstruction techniques. Flow cytometry coupled with ROS inhibition was employed to analyze denervated muscles and to elucidate the relationship between neutrophils and ROS. Using transcriptomic analysis combined with flow cytometry, we identified the subtypes and corresponding specific chemokines present in denervated muscle tissue. Genetically modified mice treated with neutrophil blockers were examined to demonstrate that neutrophils slow the process of atrophy in denervated muscle. Finally, P53 knockout (KO) mice were examined to confirm that P53 mediates neutrophil apoptosis. The results of our study revealed the relatively complete course of neutrophils in denervated muscle and identified neutrophils as a potential therapeutic target in the treatment of denervated muscle atrophy.

## Materials and Methods

### Antibodies and reagents

Anti-CD45 antibody was obtained from BD. FVS was obtained from eBioscience. TUNEL was obtained from Beyotime. Anti-Ly6g (1A8), anti-CD11b, anti-CD182 (CXCR2) and anti-CD192 (CCR2) antibodies were obtained from BioLegend. PECAM-1 (390) was obtained from Thermo Fisher Scientific. DNase I was obtained from Solarbio. Diphtheria toxin and fluorescein isothiocyanate (FITC)-albumin were obtained from Sigma–Aldrich. Acetylcysteine (L-NAC) was obtained from Absin.

### Experimental animals

Male Ly6g-DTR-GFP mice (*N* = 6) and P53 KO mice (*N* = 18) aged 6–weeks and weighing 22–25 g were purchased from the Model Organisms Center (Shanghai, China). Male C57BL/6 mice (*N* = 137) aged 6–8 weeks and weighing 22–25 g were purchased from Vital River Laboratory Animal Technology Co. (Zhejiang, China). Mice were housed in standard cages in a room at 23 °C and 50% relative humidity on a 12-h:12-h light:dark cycle. Mice in both denervated groups were anesthetized and subjected to unilateral sciatic nerve transection^37^. Briefly, after deep anesthetization, a 0.5-cm-long portion of the sciatic nerve in the right hind leg of each mouse was resected; the two nerve ends were buried in the muscles, and the incision was closed using 4-0 absorbable sutures. The mice were randomly assigned to experimental groups for analysis at specific time points after denervation. The animal study was reviewed and approved by the Institutional Animal Care and Use Committee of Huashan Hospital, Fudan University.

### Cell preparation and flow cytometry

#### Preparation of gastrocnemius muscle single-cell suspensions

Gastrocnemius muscle tissue was rinsed with phosphate-buffered saline (PBS), placed in a 6-cm petri dish, and cut into small pieces (2-4 mm). DNase I (1 mg/mL) was mixed with 1640 medium, and 5 ml was added to the tissue pieces. Tissue block solution was added to a gentleMACS C tube, and 5 μl of type II collagenase was added. The tissue block was then placed in the gentleMACS C tube with the enzyme solution, the tube was placed in the casing of the gentleMACS tissue processor, and the heating module was inserted. The 37C-mr-SMDK-1 program was selected for dissociation, and after the program finished running, the C tube was removed and briefly centrifuged to pellet the tissue debris. The resuspended cells were filtered with a 70-μm filter, and the resulting cell suspension was collected in 50-ml test tubes. The filters were rinsed with 10 ml of RPMI 1640 medium, the rinse medium with resuspended cells was centrifuged for 5 min, and the supernatant was discarded. The resuspended cells were then counted and stained.

#### Preparation of peripheral blood monocyte suspensions

The mouse was anesthetized with isoflurane. A 1-ml syringe was connected to a No. 7 needle, the syringe was inserted into the inner canthus, and it was rotated toward the fundus. The insertion depth was 2~3 mm. Due to the blood pressure, the blood flows into the tube by itself, and the needle can be pulled out. To prevent puncture hole bleeding, gauze was used to oppress the eyeball to achieve hemostasis. Blood (0.2 ml) was collected from the mice at one time. A total of 50 μl of anticoagulant was added to the mouse red blood cell lysate, the mixture was diluted to 1 ml (BioLegend, Cat # 420301), it was added to the blood sample, and the sample was incubated at room temperature and away from light for 15 min. The lysate was then centrifuged at 4 °C at 400 × *g* for 5 min, and the sample was observed for the presence of red blood cells. If red blood cells were observed, 1 ml of red blood cell lysate at room temperature was added while avoiding light and centrifuged at 4 °C for 5 min. Next, 3 ml of PBS was added to the resuspended cells and centrifuged at 4 °C for 5 min. Finally, the cells were resuspended and counted and stained.

#### Staining and detection

FVS dye (1 μl) was added to a suspension of single cells (1 ml; 3×10^6^ cells) slowly and evenly using a liquid transfer gun, and the suspension was then incubated at room temperature away from light for 30 min. Next, 3 ml of PBS was added to resuspend the cells, and the sample was centrifuged at 4 °C at 400 × *g* for 5 min, after which the supernatant was discarded. The cells were resuspended in PBS and then incubated with 5 μl of CD45-APC-Cy7, Ly6G-PE, CD11b-PEcy7, CXCR2-APC, or CCR2-FITC at room temperature in the absence of light. After incubation, 3 ml of PBS was added to resuspend the sample, which was centrifuged at 4 °C at 400 × *g* for 5 min, and the supernatant was discarded. Prior to detection, 200 μl of PBS was added to each tube. For TUNEL analyses, 3 ml of PBS was added to resuspend the sample, which was centrifuged at 4 °C at 400 × g for 5 min, and the supernatant was discarded. One milliliter of broken membrane buffer was added to resuspend the cells, and the sample was incubated at room temperature in the absence of light for 45 min. Three milliliters of broken membrane buffer was added to resuspend the sample, which was centrifuged at 4 °C at 400 × *g* for 5 min, and the supernatant was discarded. Then, 100 μl of broken membrane buffer was added to resuspend the cells, and the sample was incubated with Intramembrane factor at room temperature in the absence of light for 30 min. Three milliliters of broken membrane buffer was added to resuspend the sample, which was centrifuged at 4 °C at 400 × *g* for 5 min, and the supernatant was discarded. Prior to detection, 200 μl of broken membrane buffer was added to each tube. The data were analyzed using FlowJo software.

### Detection of ROS

For ROS detection, 3 ml of PBS was added to 1 ml of single-cell suspension (3 × 10^6^ cells) to resuspend the cells. The cells were centrifuged at 4 °C at 400 × *g* for 5 min, and the supernatant was discarded. Next, 1 ml of 2’,7’-dichlorodihydrofluorescein diacetate (DCFH-DA) solution with a concentration of 10 μmol/L, which was diluted with the serum-free medium at a ratio of 1:1000, was added to the cell precipitate. The sample was incubated at 37 °C for 30 min with mixing by inverting the tube every 3–5 min so that the probe remained in contact with the cells. The cells were then centrifuged and washed with serum-free cell culture medium three times by centrifugation at 4 °C at 400 × *g* to fully remove the DCFH-DA that did not enter the cells. Next, 200 μl of the serum-free medium was added to resuspend the cells. The cells were then examined by flow cytometry, and the data were analyzed using FlowJo software.

### ROS inhibition

Before denervation, mice were injected intravenously (tail vein) with solvent control (saline) or L-NAC at a dose of 1.1 mmol/kg/d for 3 days (including the day of denervation).

### Spinning-disk confocal intravital microscopy (SD-IVM)

Mice were subjected to sciatic nerve transection and exposure of the ipsilateral gastrocnemius muscle under anesthesia with isoflurane; inhaled anesthesia was maintained throughout the subsequent intravital imaging experiment. Mouse body temperature was maintained using a heating pad. Gastrocnemius muscle intravital imaging experiments were performed using a spinning-disk confocal microscope (Zeiss Axio Observer Z1 basic stand inverted scope equipped with a Yokogawa CSU-X spinning disk unit and an EMCCD camera [Hamamatsu]). Samples were excited with 488-nm and 633-nm laser lines, and emission was collected using a 10×/0.3 and 25×/0.8 objective with standard emission filter sets (Semrock). Images were acquired using ZEN acquisition software (Zeiss). The muscle microvasculature was visualized by intravenous infusion of 5 µg of anti-PECAM-1. Neutrophils inside the muscle microvasculature and tissue were visualized via topical application of fluorescently labeled anti-Ly6G (1A8) antibody.

### Slice digital scanning and 3D-view reconstruction

#### Serial slices and immunofluorescence

Gastrocnemius muscle samples were fixed in 4% paraformaldehyde and embedded in paraffin. The samples were completely cut into 50 pieces with a thickness of 6 µm and subjected to double immunostaining. Paraformaldehyde-fixed muscles were incubated with the primary antibody, followed by incubation with the secondary antibody and subsequent mounting with DAPI. After staining and immunofluorescence, the 50 serial slices were quickly placed under the microscope lens of the slice scanner for continuous scanning and imaging, and whole full-field digital slices were generated by seamless splicing using the control software system.

#### Panoramic 3D-view reconstruction

Images were stacked using the 3D-View software voloom (Bitplane, Switzerland) to form a three-dimensional map that could be cut and rotated using related software. The file contained all relevant tissue information for each section. A Panoramic MIDI scanner (3D HISTECH, Hungary) was used.

#### Imaris 3D-view reconstruction

Within the software, ruler size was selected in the Display window, and the Edit button was used to select the size of the captured image in the Geometry tab. In the 3D-View view, the Animation button was used to set the 3D view, and the image effect was adjusted by selecting Display and varying the min/max/gamma values. The display color of the image was selected using the channel color feature. The appropriate number of frames was selected according to the desired video display, and video nodes were then added. The added nodes were modified or added as necessary using the rotations program by clicking on the right triangle for playback and saving the video.

### RNA-seq

Total RNA was extracted from muscle tissue samples using the miRNA Isolation Kit (mirVana; Thermo Fisher Scientific, Waltham, MA, United States; AM1561) according to the manufacturer’s protocol. The RNA integrity was evaluated using the Agilent 2100 Bioanalyzer (Agilent Technologies, Santa Clara, CA, United States), and samples with an RNA integrity number ≥ 6 were retained for analysis. Then, the libraries were constructed using the TruSeq Stranded mRNA LT Sample Prep Kit (Illumina, San Diego, CA, USA) according to the manufacturer’s instructions and sequenced on the Illumina HiSeq X Ten platform, generating 125/150-bp paired-end reads. Index-coded sample clustering was performed using the TruSeqPE Cluster Kit v3-cBot-HS (Illumina) on a cBot Cluster Generation System according to the manufacturer’s protocol. The Illumina HiSeq X platform was used to sequence the library preparations; 125/150-bp paired-end reads and 50-bp single-end reads were generated.

### Bioinformatic analysis

#### Quality control and mapping

Raw data (raw reads) were processed using Trimmomatic^38^. Reads containing poly-N and those of low quality were removed to obtain clean reads, which were mapped to the reference genome using hisat2^39^.

#### Identification of Differentially Expressed Genes (DEGs) and Gene Ontology (GO) and Kyoto Encyclopedia of Genes and Genomes (KEGG) pathway enrichment analyses

The fragments per kilobase of transcript per million mapped reads value^40^ of each gene was calculated using cufflinks^41^, and the read counts of each gene were obtained with htseq-count^42^. Differentially expressed genes (DEGs) were identified using the DESeq package of R software with the estimateSize Factors and nbinomTest functions. A *P* value < 0.05 and fold change > 2 or < 0.5 were set as the thresholds for significantly different expression. Hierarchical cluster analysis of DEGs was performed to explore gene expression patterns. Gene Ontology (GO) enrichment and Kyoto Encyclopedia of Genes and Genomes (KEGG) pathway enrichment analyses of DEGs were performed using R based on the hypergeometric distribution^43^.

### Quantitative real-time PCR (qPCR)

The RNeasy kit (Qiagen, Valencia, CA, United States) was used to extract total RNA from gastrocnemius muscle. cDNA was synthesized using a first-strand cDNA synthesis kit with oligo dT primers (Invitrogen, Carlsbad, CA, United States) and was then used for quantitative real-time PCR (qPCR) (MJ Research, Waltham, MA, United States). The thermal cycling conditions were as follows: 94 °C for 5 min; 35 cycles at 94 °C for 30 s, 55 °C for 45 s, and 72 °C for 1 min; and 72 °C for 5 min. The relative expression level of the target gene was calculated using the cycle threshold (Ct) value. CXCL1 expression levels were normalized to GAPDH levels. Primer sequences were as follows: R, 5’-CTG AAC AAG CAG AAC TGA ACT AC-3’, F, 5’-GTA ACG GAG AAA GAA GAC AGA CT-3’.

### Neutrophil depletion

Neutrophil depletion was performed via intraperitoneal administration of 200 µg of anti-Ly6G (1A8, Bio X Cell) antibody 24 h prior to injury induction for the SD-IVM experiment^12^. To evaluate neutrophil function in denervated muscle, C57BL/6 mice were injected intraperitoneally with an initial 400 μg followed by 100 μg three times weekly for anti-Ly6G (1A8, Bio X Cell) to deplete neutrophils. Neutrophils were depleted in Ly6g-DTR-GFP mice by intraperitoneal injection of 10 μg/g body weight of diphtheria toxin (DT) three times weekly^44^.

### Wet weight

At 7 and 14 days after denervation, mice were anesthetized, and the gastrocnemius muscles of both the left and right hind legs were removed, washed with saline, and then weighed. The ratio of muscle weight loss was defined as the weight of the contralateral side minus the muscle weight of the nerve injury side divided by the weight of the contralateral side. The muscle samples were stored in 4% paraformaldehyde at −80 °C until use.

### Hematoxylin–eosin (HE) staining

Gastrocnemius muscle samples from mice were fixed in 4% paraformaldehyde and embedded in paraffin. The samples were cut at a thickness of 5 µm, and the sections were stained with HE (Beyotime, Shanghai, China) to evaluate histopathologic changes. The mean area, diameter, and density of myofibers were determined by blinded analysis using Image-Pro Plus 6.0 software (National Institutes of Health, Bethesda, MD, USA) from six randomly captured images per mouse under each experimental condition. An EclipseCi-L camera microscope was used to select the target area of the tissue for 400x imaging so that the tissue could fill the whole field of view as much as possible to ensure that the background light of each photo was consistent. After completion of imaging, Image-Pro Plus 6.0 analysis software was used to count the number of muscle fibers in three visual fields in each slice (mm²), to measure the diameter of five muscle fibers (mm), and to calculate the average muscle fiber area (mm²) (equal to the total area of muscle fibers/number of muscle fibers) and the muscle fiber density (equal to the number of muscle fibers/area of the visual field).

### Statistical analysis

Measurement variables are presented as the mean ± standard error of the mean (SEM), and categorical variables are presented as percentages. Differences between 2 groups were evaluated using the *χ*^*2*^ test. For comparisons among multiple groups, one-way analysis of variance (ANOVA) tests were conducted. Differences at different time points were evaluated by using the Friedman test. Statistical analyses were performed using SPSS software, v17.0 (SPSS Inc., Chicago, IL, USA). *P* values < 0.05 were considered statistically significant.

## Results

### Neutrophils were detected in denervated muscles after peripheral nerve injury

**Fig. 1:**
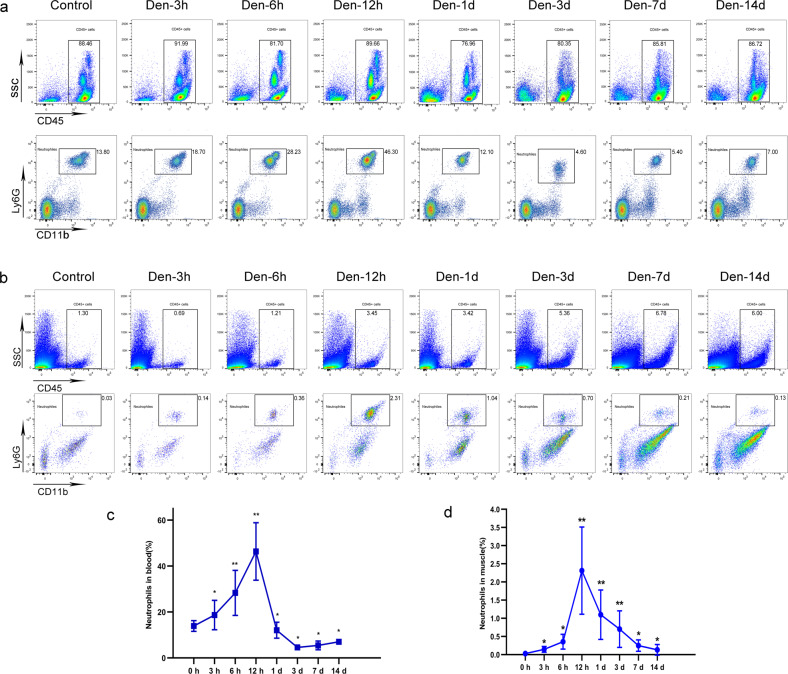
Dynamic changes in neutrophils in peripheral blood and denervated muscle. **a** Flow cytometry analysis of neutrophils in control WT peripheral blood and blood at 3 h, 6 h, 12 h, 1 day, 3 days, 7 days, and 14 days after denervation. **c** Quantification of the dynamic changes in neutrophil populations as a percentage of peripheral blood cells. ^*^P < 0.05 and ^**^P < 0.01 versus 0 h (*n* = 4/group). **b** Flow cytometry analysis of neutrophils in control WT gastrocnemius muscle and muscle 3 h, 6 h, 12 h, 1 day, 3 days, 7 days, and 14 days after denervation. **d** Quantification of the dynamic changes in neutrophil populations as a percentage of cells in denervated gastrocnemius muscle. ^*^P < 0.05 and ^**^P < 0.01 versus 0 h (*n* = 4/group). WT wild type.

To comprehensively investigate the dynamic changes in mouse neutrophil populations in vivo after peripheral nerve injury, we first performed time-course flow cytometry analyses of neutrophils in both peripheral blood and denervated gastrocnemius muscle (duration 3 h, 6 h, 12 h, 1 day, 3 days, and 7 days; Fig. 1a, b). A sustained increase in the number of neutrophils was observed in both the peripheral blood and gastrocnemius muscle throughout the early period after denervation (<24 h after denervation). The number of neutrophils peaked 12 h after denervation and returned to a lower stable level after 3 days (Fig. 1c, d).

### Neutrophils were activated by ROS generated after denervation

**Fig. 2:**
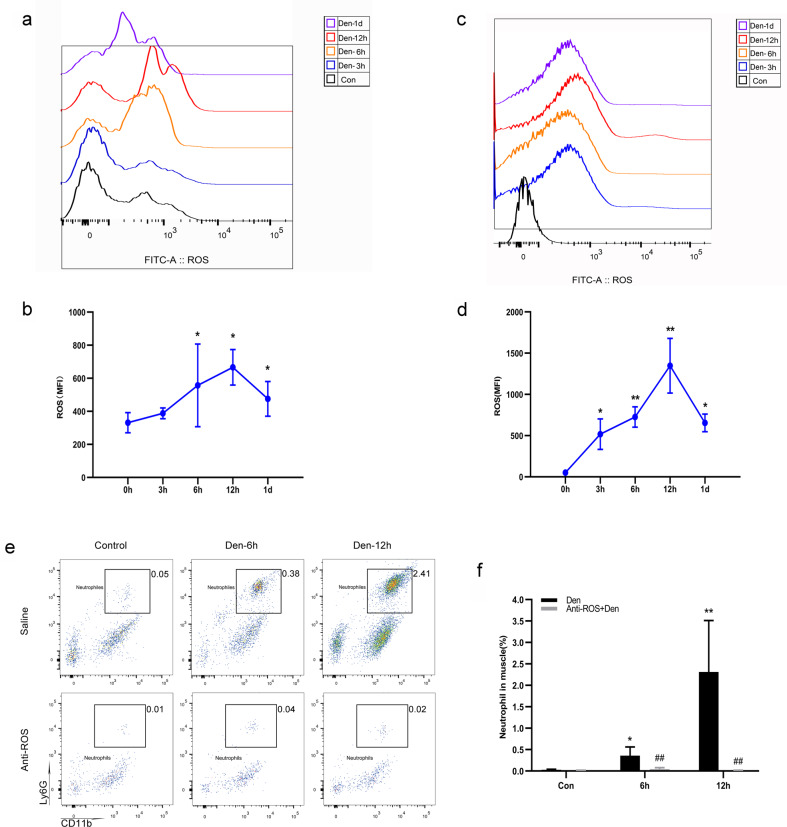
Increased ROS levels activate neutrophils after denervation. **a** Flow cytometry analysis of ROS in control WT peripheral blood and blood at 3 h, 6 h, 12 h, and 1 day after denervation. **b** Dynamic changes in ROS in peripheral blood. ^*^P < 0.05 versus 0 h (*n* = 4/group). **c** Flow cytometry analysis of ROS in control WT gastrocnemius muscle and muscle 3 h, 6 h, 12 h and 1 day after denervation. **d** Dynamic changes in ROS in denervated gastrocnemius muscle. ^*^P < 0.05 and ^**^P < 0.01 versus 0 h (*n* = 4/group). **e** WT mice were treated with a ROS inhibitor at the optimal dose (200 μg/mouse) or with saline before and after denervation. Flow cytometry analysis of neutrophils in control WT gastrocnemius muscle and muscle 6 and 12 h after denervation. **f** Quantification of neutrophils as a percentage of cells in denervated gastrocnemius muscle. ^*^P < 0.05 and ^**^P < 0.01 versus control; ^##^*P* < 0.01 versus Den-6h and Den-12h (*n* = 4/group). ROS reactive oxygen species, WT wild type.

We next examined the initiator of neutrophil activation using time-course flow cytometry analyses of ROS in both peripheral blood and denervated gastrocnemius muscle of mice to characterize the dynamic changes in ROS in vivo (duration 3 h, 6 h, 12 h and 1 day; Fig. 2a, b). In both peripheral blood and gastrocnemius muscle, increased ROS production was sustained throughout the early period after denervation (< 24 h after denervation) and peaked at 12 h (Fig. 2c, d), similar to the trend observed with neutrophils. To confirm the relationship between increased ROS and neutrophil numbers, we examined whether ROS blockade prevents the increase in neutrophil numbers. Twenty-four WT mice were treated with L-NAC or saline before and after denervation, and neutrophils were analyzed by flow cytometry (Fig. 2e). In ROS-inhibited mice, a significant reduction in the number of neutrophils was observed 6 h and 12 h post-denervation in comparison with control saline-treated mice (Fig. 2f), indicating that ROS production plays a role in neutrophil activation.

### Dynamic behavior of neutrophils in blood vessels of denervated muscle

**Fig. 3:**
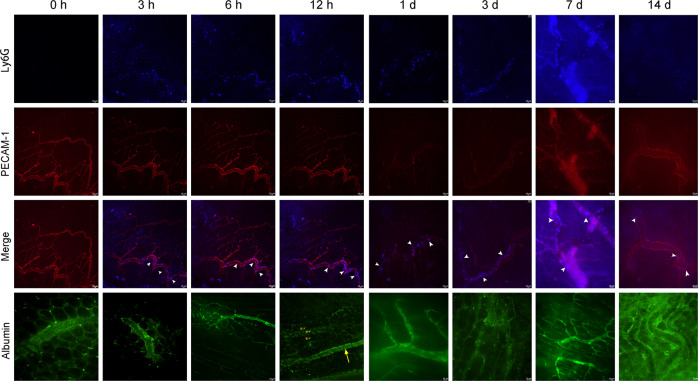
Dynamic behavior of neutrophils inside blood vessels of denervated muscle. Representative images of the accumulation of neutrophils (Ly6G: blue) and trafficking from the peripheral blood circulation to the blood vessels (PECAM-1: red) of denervated muscle in response to sciatic nerve injury in the control and 3 h, 6 h, 12 h, 1 day, 3 days, 7 days, and 14 days after denervation. Albumin (green) was injected to evaluate the patency and permeability of blood vessels, which exhibited no visible exudation. Representative images of blood vessels (large yellow arrow) and muscle fibers (small yellow arrows). Scale bars, 10 μm.

Next, both the number and dynamic behavior of neutrophils recruited from the peripheral circulation to skeletal muscle after denervation were examined in detail. In the sciatic nerve injury model, an increase in neutrophil trafficking from the peripheral circulation to blood vessels of muscle tissue occurred following denervation (duration 3 h, 6 h, 12 h, 1 day, 3 days, 7 days, and 14 days). The neutrophils rolled, crawled, and clearly accumulated in denervated muscle vessels (Fig. 3; blue particles indicated by white arrowheads), reaching a peak at 12 h (Fig. 3; Supplementary Movie 1, 2, 3, and 4). Neutrophils were constantly trafficked from the peripheral blood to the muscle vessels in the subsequent process of denervation (Supplementary Movie 5, 6, 7, and 8). This finding was consistent with the dynamic increase in neutrophils observed throughout the denervated muscle. The flow of FITC-albumin (administered intravenously after denervation) through the vessels of denervated muscle was normal compared to the control, and no FITC-albumin leaked out of the vessels within 14 days (Supplementary Movie 9, 10, 11, 12, 13, 14, 15, and 16), suggesting that the blood vessels were unobstructed and that there was no obvious exudation.

### Neutrophils migrate from blood vessels to denervated muscle tissue

**Fig. 4:**
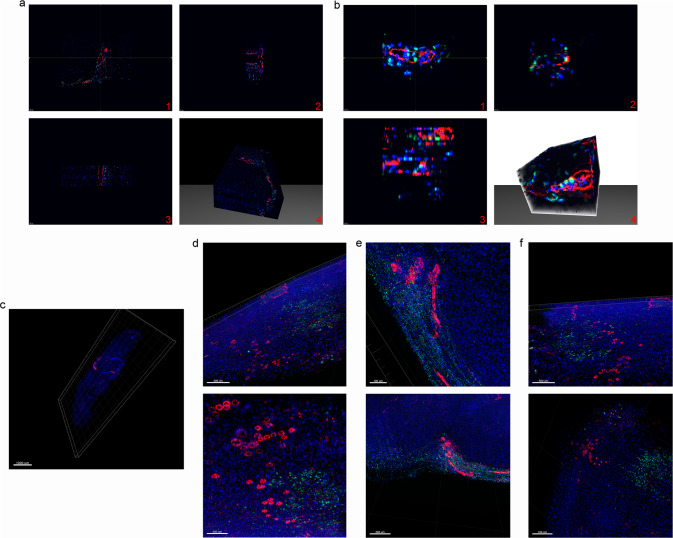
Neutrophils escape from blood vessels and migrate into denervated muscle. **a, b** Representative 3D panoramic scanning images of recruited neutrophils (green) migrating from the blood vessels (red) to denervated muscle tissue (DAPI: blue) 12 h after sciatic nerve injury. A: Scale bars, 50 μm; B: Scale bars, 10 μm. **c, d, e, f** Representative 3D-view images of Imaris reconstruction showing the separated spatial distribution of blood vessels and neutrophils in denervated muscle 12 h after sciatic nerve injury. c: Scale bar, 1000 μm; d: Scale bars, 500 μm (top), 400 μm (bottom); e: Scale bars, 100 μm (top), 300 μm (bottom); f: Scale bars, 500 μm (top), 100 μm (bottom).

We next assessed whether neutrophils escaped and migrated from vessels into the muscle tissue after denervation. Fifty consecutive sections of denervated gastrocnemius muscle were evaluated by 3D panoramic reconstruction after fluorescent staining (Figs. 4a-4; Supplementary Movie 17). The single slice showed that neutrophils were distributed in the denervated muscle tissue outside the large-diameter blood vessel (Figs. 4a-1). The same phenomenon was observed on the 3D section of the y-axis (purple line) and X-axis (green line) slices (Figs. 4a-2, 3). We also observed a similar spatial distribution inside and outside the small-diameter blood vessel (Fig. 4b; Supplementary Movie 18). After digital scanning of the slices, the spatial distribution of neutrophils and blood vessels in different visual fields of the whole gastrocnemius muscle was further verified using the Imaris 3D-view reconstruction technique (Fig. 4c; Supplementary Movie 19). Neutrophils were widely distributed in denervated muscle blood vessels and tissues from two different visual angles in three different spaces of the gastrocnemius muscle region (Fig. 4d, e, f). These results confirmed that neutrophils infiltrate denervated muscle tissue from blood vessels, which further indicates that neutrophils play a role in the pathologic changes in skeletal muscle after denervation.

### CXCL1 recruits CXCR2 neutrophils to denervated muscle

**Fig. 5:**
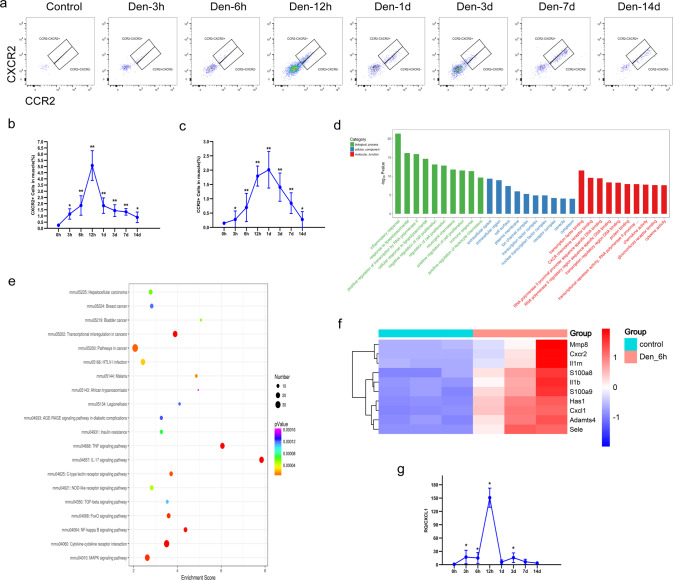
CXCL1 recruits CXCR2 neutrophils to denervated muscle. **a** Flow cytometry analysis of recruited CXCR2^+^ and CCR2^+^ neutrophils in control WT gastrocnemius muscle and muscle 3 h, 6 h, 12 h 1 day, 3 days, 7 days, and 14 days after denervation. Representative gating is shown. **b,** **c** Quantification of the dynamic changes in the CXCR2^+^ and CCR2^+^ subsets as a percentage of cells in denervated gastrocnemius muscle. ^*^P < 0.05 and ^**^P < 0.01 versus 0 h (*n* = 4/group). **d** RNA-seq identified the top 30 GO terms associated with denervated gastrocnemius muscle. **e** Top 20 KEGG pathways of DEGs. **f** Heatmap of the top 10 upregulated DEGs in gastrocnemius muscle from control WT mice and muscle 6 h after denervation. Heatmap colors indicate directionality (red: increased; blue: decreased). **g** CXCL1 mRNA expression in control WT mouse gastrocnemius muscle and muscle after denervation. ^*^P < 0.05 vs. 0 h (*n* = 4/group). DEG differentially expressed gene, WT wild type.

We further found that neutrophils in denervated muscle of mice could be divided into 2 subsets, namely, CXCR2^+^ and CCR2^+^ cells, of which CXCR2^+^ neutrophils accounted for the vast majority (Fig. 5a). The number of CXCR2^+^ neutrophils peaked 12 h post-denervation and returned to a low stable level 3 days post-denervation (Fig. 5a, b), consistent with the overall trend. In contrast, the number of infiltrating CCR2^+^ cells peaked 1 day post-denervation and remained elevated at 7 days post-denervation (Fig. 5a, c). To investigate the infiltration of CXCR2^+^ cells during denervation, the gastrocnemius muscle transcriptome data for 6 h post-denervation were mined, which revealed that (1) the top 30 enriched Gene Ontology (GO) terms associated with the identified differentially expressed genes (DEGs) according to the threshold *P* value (*P* < 0.05) included molecular functions, such as “CXCR chemokine receptor binding” (Fig. 5d); (2) the top 20 enriched Kyoto Encyclopedia of Genes and Genomes (KEGG) pathways of the DEGs included “cytokine–cytokine receptor interaction” (Fig. 5e); and (3) CXCL1, a specific chemokine for CXCR2, was among the top 10 upregulated genes (Fig. 5f). Quantitative reverse-transcription polymerase chain reaction assays showed that muscle CXCL1 expression increased significantly 3 h post-denervation, with peak CXCL1 expression at 12 h (Fig. 5g). Collectively, these data suggest that a specific cluster of CXCR2^+^ neutrophils is recruited to denervated muscle tissue by CXCL1.

### Neutrophils contribute to the delay in denervated muscle atrophy

**Fig. 6:**
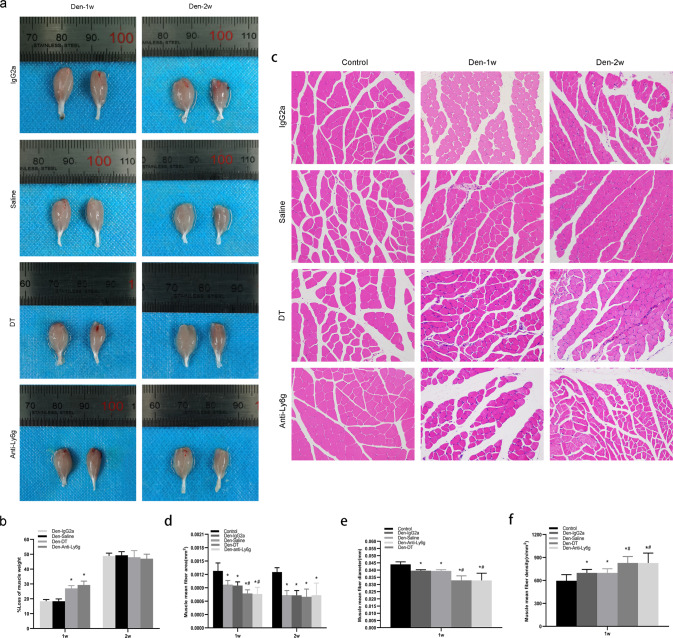
Neutrophils attenuate denervated muscle atrophy during the first week. Ly6g-DTR-GFP mice were injected with DT at the optimal dose (10 μg/g body weight/mouse, 3 times/week) before and after denervation. WT mice were treated with anti-Ly6g clone 1A8 at the optimal dose (200 μg/mouse, 3 times/week) after denervation. **a, b** The appearance and loss of gastrocnemius muscle weight at 1 week and 2 weeks post-denervation. ^*^P < 0.05 versus Den-Saline or Den-IgG2a (*n* = 6/group). **c, d, e, f** HE staining of muscle tissue (200×) and mean ± SEM fiber area, mean fiber diameter, and mean fiber density showing muscle atrophy that was ameliorated by neutrophils. ^*^P < 0.05 versus Control; ^#^*P* < 0.05 versus Den-Saline or Den-IgG2a (*n* = 6/group). WT, wild-type, Ly6g-DTR-GFP mice, heterozygous mice with ly6g gene knock-in IRES-DTRGFP; DT diphtheria toxin.

To explore the function of neutrophils in a model of denervation injury, mice were treated with the neutrophil inhibitor DT (diphtheria toxin) and anti-Ly6g clone 1A8. Denervation-induced loss of muscle weight was significantly aggravated 7 d after injection of DT and anti-Ly6g clone 1A8 (Fig. 6a, b). In addition, we also assessed the fiber after staining muscle sections with H&E dyes (Fig. 6c) and found that the mean fiber area (Fig. 6d) and mean fiber diameter of the gastrocnemius muscle (Fig. 6e) were smaller, and the mean fiber density of the gastrocnemius muscle (Fig. 6f) was higher than that of saline-injected mice 7 d post-denervation. However, there was no change versus 14 d post-denervation. These results suggest that neutrophils delay atrophy of denervated muscle in the first 7 days.

### Neutrophils undergo apoptosis via P53 with the development of muscle atrophy

**Fig. 7:**
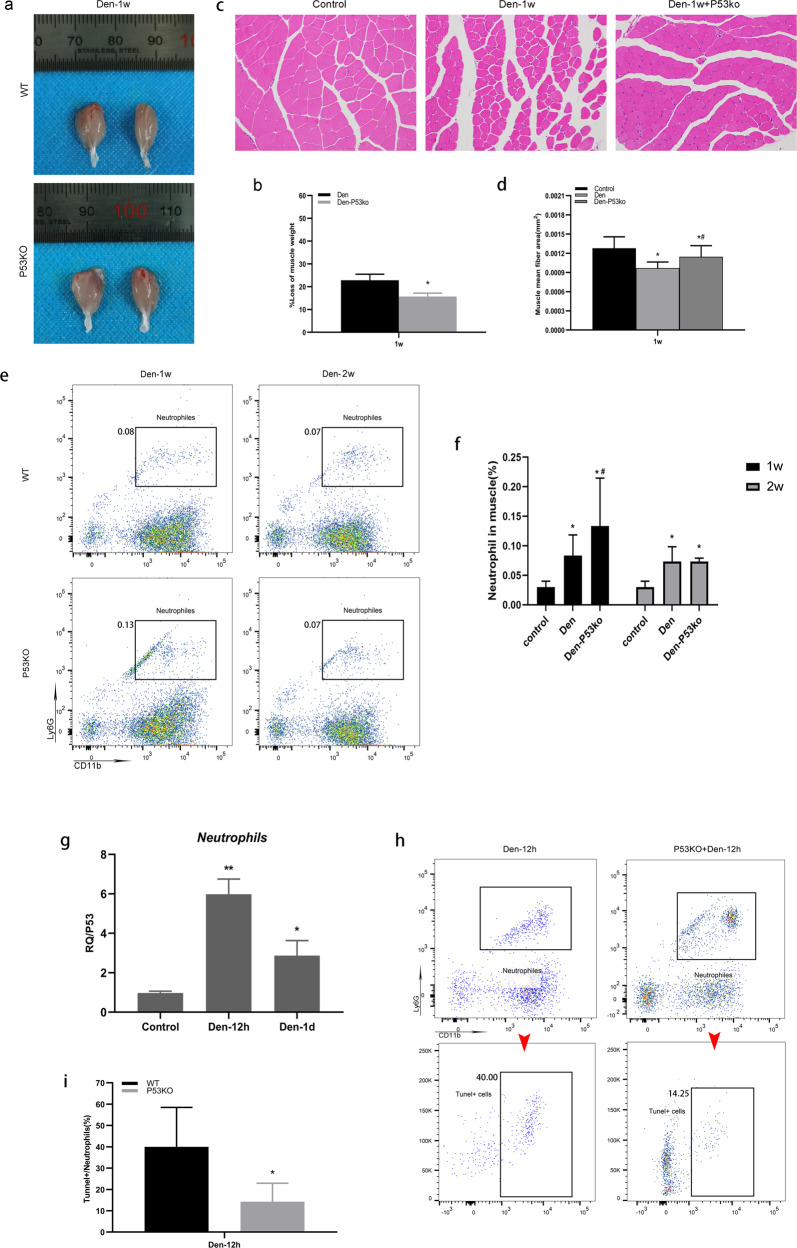
Neutrophils undergo apoptosis via P53 with the development of muscle atrophy. **a, b** The appearance and weight loss of the gastrocnemius muscle at 1 week post-denervation. ^*^P < 0.05 versus Den (*n* = 6/group). **c, d** HE staining of muscle tissue (200×) and the mean ± SEM fiber area; sections show that muscle atrophy was reduced following P53 knockout. ^*^P < 0.05 versus Control; ^#^*P* < 0.05 versus Den (*n*=6/group). **e** Flow cytometry analysis of neutrophils in control WT and P53 KO gastrocnemius muscle 1 week and 2 weeks after denervation. **f** Quantification of neutrophils as a percentage of cells of denervated gastrocnemius muscle. ^*^P < 0.05 versus Control; ^#^*P* < 0.05 versus Den (*n* = 4/group). **g** Level of *P*53 was measured by real-time polymerase chain reaction in neutrophils. At Control, Den-12 and Den-1d groups, the neutrophils were isolated by flow sorting, respectively. **P < 0.01 versus Control, *P < 0.05 versus Control. **h** Flow cytometry analysis of neutrophils and corresponding TUNEL-positive neutrophils in WT and P53 KO gastrocnemius muscle 12 h after denervation. **i** Quantification of TUNEL-positive cells as a percentage of neutrophils in denervated gastrocnemius muscle. ^*^P < 0.05 versus WT (Den-12h) (*n* = 4/group). WT wild type, KO knockout.

Denervation-induced loss of muscle wet weight was significantly attenuated (Fig. 7a, b), and the mean fiber area of the gastrocnemius muscle was greater in P53 KO mice than in WT mice (Fig. 7c, d). A significant increase in the number of neutrophils in P53 KO mice was observed 7 d post-denervation in comparison with WT mice, whereas there was no difference in the number of neutrophils 14 d post-denervation (Fig. 7e, f). These findings indicate that both P53 KO and neutrophils delay denervated muscle atrophy during the first 7 d and that the decrease in the number of neutrophils is probably mediated by upregulation of P53. To confirm these results, we first separated the neutrophils from denervated muscles by flow cytometry and found that compared with that in the control group, the level of p53 in neutrophils increased significantly and peaked at 12 h post-denervation (Fig. 7g). Next, we examined the number of neutrophils in P53 KO mice and found a significant increase at 12 h post-denervation in comparison with WT mice, but a lower percentage of TUNEL-positive apoptotic neutrophils was observed compared with WT mice (Fig. 7h, i). The results of the one-step apoptosis assay of TUNEL cells showed that neutrophils peaked at 12 h and then underwent apoptosis via the p53-mediated apoptotic pathway within 7 days after denervation. P53 KO markedly suppressed neutrophil apoptosis.

## Discussion

Here, we visualized a previously unrecognized fate of neutrophils in denervated muscle. When peripheral nerve injury initiates a systemic stress response, ROS levels in peripheral blood and denervated muscle increase significantly, thereby facilitating an increase in the proportion of neutrophils in the peripheral blood. We propose that depending on the activation of ROS, CXCR2^+^ neutrophils—guided by a specific chemokine, CXCL1—regularly traffic from the circulation to denervated muscle blood vessels, migrate to muscle tissue, delay atrophy, and then undergo apoptosis.

Previous basic and clinical studies have suggested that neutrophils are major participants in early inflammatory processes and can be swiftly deployed to sites of damage or infection^45–47^. However, to date, only a few inflammation-related molecules have been found in denervated muscle^48,49^. Although denervated muscle cells themselves are not directly harmed or infected and no local pathogens attract neutrophils during denervation, we demonstrated the dynamic changes in the number of neutrophils in the circulation and in muscle over time after denervation. Neutrophil numbers increased slightly before 6 h and then increased rapidly, peaking at 12 h. Only a small percentage of neutrophils remained over the long term. Hence, it is conceivable that local denervation injury leads to an increase in the recruitment of neutrophils from the circulation to corresponding denervated muscle areas.

Previous intravital imaging experiments of infection^50,51^ and sterile injury models^12,14^ provided visual evidence of neutrophil migration to target sites. To obtain the most convincing evidence of neutrophil migration pertaining to denervated muscle atrophy, real-time intravital imaging experiments were performed using spinning-disk confocal microscopy in the present study. To our knowledge, we provide the first conclusive evidence that neutrophils directly and clearly appear in denervated muscle vessels in the absence of direct muscular injury. In addition, Wang et al.^12^ showed that due to the lack of patency of the vessels within sites of direct injury, FITC-albumin leaked out of the vessels at the border of the injury, and no FITC-albumin moved into the collapsed vessels inside the necrotic area. Conversely, our results demonstrate that the vessels of denervated muscle always remain patent during denervation. Furthermore, it is worth noting that our study demonstrated omni-directional 3D-view findings, namely, we revealed the spatial distribution of neutrophils in denervated muscles, particularly the spatial relationship with blood vessels. Our reconstructed data clearly indicate that neutrophils can escape from the blood and be distributed simultaneously in the vessels and muscle tissue.

An imbalance between the generation of ROS and the cell’s ability to metabolize these factors causes oxidative stress^52,53^. Previous studies have suggested that an enhanced generation of ROS is a common factor in the mechanism underlying denervation-induced atrophy^21,23^. We and others^21,23^ discovered that ROS production increases significantly in denervated muscle, as does ROS production in the circulation. Interestingly, the change in the trend of ROS generation was consistent with the increase in the number of neutrophils. Previous studies have shown that neutrophils can be activated directly or indirectly by ROS after acute injury or infection^16,54,55^. Therefore, blocking ROS generation by administration of a ROS inhibitor in our mouse denervated muscle model led to a sharp decrease in the number of neutrophils. We thus demonstrate that ROS are an important regulator of neutrophil activation after denervation.

Specific pathogens and inflammatory mediators, such as chemokines, are essential for neutrophil recruitment following infection^56,57^ or tissue damage^58^. However, denervated muscle cannot be classified in the same way as classical local lesions that are subjected to direct damage are classified^12^. As neutrophils can migrate to denervated muscle, there must be some specific chemoattractant. Previous research has found that neutrophils in mouse blood can be divided into two subsets: Ly6G^high^CXCR2^+^ and Ly6G^low^CCR2^+^^59^. Based on our previous flow cytometry analysis of neutrophils, we further divided neutrophils into two subtypes according to the cell markers CXCR2 and CCR2. Surprisingly, we found that CXCR2^+^ neutrophils account for the vast majority and play a dominant role in the early period after denervation. In the present work, we used high-throughput sequencing to compare the gene expression profiles of denervated–6 h and normal gastrocnemius muscle. We identified 678 DEGs between denervated and nondenervated muscles, including 357 upregulated and 321 downregulated genes. Most of the enriched GO terms were directly associated with biological processes and molecular functions of neutrophils and chemotaxis, such as “inflammatory response,” “neutrophil chemotaxis,” “CXCR chemokine receptor binding,” and “chemokine activity.” The KEGG pathway analysis showed that the DEGs were heavily enriched in “IL-17 signaling pathway” and “cytokine–cytokine receptor interaction.” The IL-17 signaling pathway is reportedly a major chemotactic signaling pathway of neutrophils during inflammatory injury^60,61^. Studies of cytokine–cytokine receptor interactions primarily involve binding between chemokines and receptors^62^. Hence, numerous pathways related to neutrophils and chemotaxis were enriched in our study. Previous studies have shown that the CXC subfamily of chemokines functions as an important link in cytokine–cytokine receptor interaction pathways^62,63^. Additionally, some other studies have shown that sequential CXCR2-dependent chemokine engagement involving CXCL1 and CXCL2 can induce neutrophil recruitment into tissues^59,64^. Consistently, we show here that CXCL1, as one of the top 10 upregulated DEGs in denervated–6 h muscle that acts as a specific chemokine for CXCR2, recruits CXCR2^+^ neutrophils to denervated muscle.

Accumulating evidence supports the existence of distinct neutrophil subsets that play diverse roles in injury, infection, and cancer immunology^26,65,66^. Therefore, treatments that directly reduce the number of neutrophils regardless of the specific phenotype may inadvertently promote damage^67^. Furthermore, other studies have shown that neutrophils protect against sterile injury by removing necrotic fragments and mediating the rebuilding of blood vessels^12,14^. In our study of denervated muscle atrophy, therefore, we focused on the roles of specific clusters of neutrophils that could be more clearly targeted. Notably, we demonstrate that ROS-activated CXCR2^+^ neutrophils recruited by CXCL1 delayed denervated muscle atrophy within the first week. This finding is in line with our result that most neutrophils appear within 1 week. However, the underlying mechanism by which neutrophils delay muscle atrophy remains unclear.

Apoptosis is considered a control point that limits the number of neutrophils at sites of inflammation and pushes ongoing inflammation toward resolution^13,14^. Previous studies have found that a variety of molecules promote or inhibit neutrophil apoptosis^35,36^. P53 is a tetrameric transcription factor that is heavily regulated by post-transcriptional modifications and was initially identified as a tumor suppressor gene^68^. However, increasing evidence shows that it halts cell proliferation and induces apoptosis^69,70^. P53-mediated signaling is a classic signaling pathway regulating apoptosis^71^. Moreover, P53 reportedly modulates the expression of tripartite motif-containing 63, which regulates the proteasomal degradation of structural muscle proteins, particularly myofibril components^1,72^. However, our results demonstrate that P53 KO delays muscle atrophy, which is consistent with the function of neutrophils. It is important to note that our study demonstrates an interesting finding, namely, that P53 KO also increased the number of neutrophils within 1 week. We fully recognize that, in general, more neutrophils could better delay muscle atrophy during the early period after denervation (< 1 week). Our data found that the level of p53 in neutrophils of target muscle increased significantly after denervation. Indeed, we found that the number of TUNEL-positive neutrophils decreased significantly following P53 KO. Thus, a combination of the previously reported work mentioned above and our current results suggests a possible molecular mechanism for neutrophil apoptosis during denervation, which may occur through the p53-mediated apoptosis pathway.

**Fig. 8:**
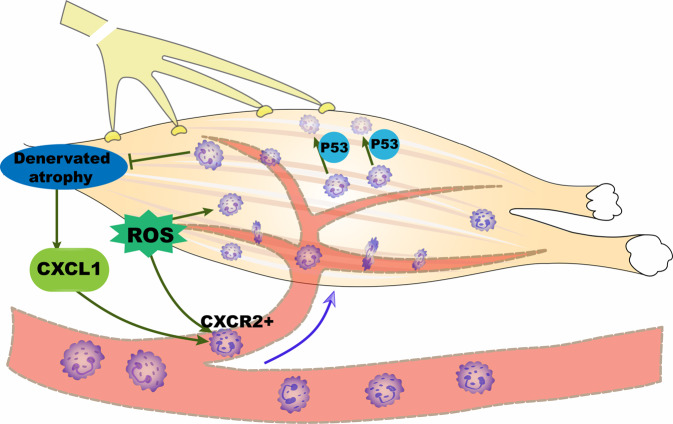
Proposed model for the fate and function of neutrophils after denervation. Denervation-induced atrophy immediately upregulates CXCL1 expression, which recruits ROS-activated CXCR2^+^ neutrophils into denervated muscle to delay skeletal muscle atrophy. Neutrophils are preprogrammed to ultimately undergo apoptosis mediated by P53.

Collectively, our findings from intravital imaging and 3D-view reconstruction analyses highlight the authenticity of neutrophil recruitment in denervated muscle and relatively clearly show the entire dynamic track of neutrophil fate after denervation (Fig. 8). Our findings suggest that neutrophils function in immune surveillance to play a protective role in denervated muscle atrophy. Understanding and probing how neutrophils delay muscle atrophy in the early period after denervation could lead to the development of new therapeutic strategies to manipulate disease pathogenesis.

## Supplementary information

M1---Neutrophils in blood vessels of denervated muscle (0-2 h)

M2---Neutrophils in blood vessels of denervated muscle (2-3 h)

M3---Neutrophils in blood vessels of denervated muscle (3-6 h)

M4---Neutrophils in blood vessels of denervated muscle (6-12 h)

M5---Neutrophils in blood vessels of denervated muscle (12-24 h)

M6---Neutrophils in blood vessels of denervated muscle (3 d)

M7---Neutrophils in blood vessels of denervated muscle (7 d)

M8---Neutrophils in blood vessels of denervated muscle (14 d)

M9--- Blood vessels of muscle remain patent 0 h after denervation

M10--- Blood vessels of muscle remain patent 3 h after denervation

M11--- Blood vessels of muscle remain patent 6 h after denervation

M12--- Blood vessels of muscle remain patent 12 h after denervation

M13--- Blood vessels of muscle remain patent 1 d after denervation

M14--- Blood vessels of muscle remain patent 3 d after denervation

M15--- Blood vessels of muscle remain patent 7 d after denervation

M16--- Blood vessels of muscle remain patent 14 d after denervation

M17--- Representative 3D panoramic scanning images of muscle 12h after denervation (big-diameter blood vessel)

M18--- Representative 3D panoramic scanning images of muscle 12h after denervation (small-diameter blood vessel)

M19--- Representative 3D-view images of Imaris of muscle 12h after denervation
